# Stereodynamical Effects by Anisotropic Intermolecular Forces

**DOI:** 10.3389/fchem.2019.00390

**Published:** 2019-05-31

**Authors:** Daniela Ascenzi, Mario Scotoni, Paolo Tosi, David Cappelletti, Fernando Pirani

**Affiliations:** ^1^Dipartimento di Fisica, Università di Trento, Trento, Italy; ^2^Dipartimento di Chimica, Biologia e Biotecnologie, Università di Perugia, Perugia, Italy

**Keywords:** alignment, orientation, stereo-dynamics, ion-molecule reactions, astrochemistry

## Abstract

Electric and magnetic field gradients, arising from sufficiently strong anisotropic intermolecular forces, tend to induce molecular polarization which can often modify substantially the results of molecular collisions, especially at low rotational temperatures and low collision energies. The knowledge of these phenomena, today still not fully understood, is of general relevance for the control of the stereo-dynamics of elementary chemical-physical processes, involving neutral and ionic species under a variety of conditions. This paper reports on results obtained by combining information from scattering, spectroscopic and reactivity experiments, within a collaboration between the research groups in Perugia and Trento. We addressed particular attention to the reactions of small atomic ions with polar neutrals for their relevance in several environments, including interstellar medium, planetary atmospheres, and laboratory plasmas. In the case of ion-molecule reactions, alignment/orientation is a general phenomenon due to the electric field generated by the charged particle. Such phenomenon originates critical stereo-dynamic effects that can either suppress (when the orientation drives the collision complex into non-reactive or less reactive configurations), or enhance the reactivity (when orientation confines reagents in the most appropriate configuration for reaction). The associated rate coefficients show the propensity to follow an Arrhenius and a non-Arrhenius behavior, respectively.

## Introduction

The focus of the present work is on investigating the role of electric and magnetic field gradients, arising from anisotropic intermolecular forces, which can induce molecular polarization (*i.e*. alignment/orientation of rotational angular momentum / bond direction of a molecule along a preferential axis) as a consequence of collisions with other atoms or molecules. Deep knowledge of these phenomena, today still not fully understood, is of general relevance to control the stereodynamics of elementary chemical-physical processes, occurring both in gaseous and condensed phases under a variety of conditions (Vattuone et al., 2004, 2009, 2010; Gerbi et al., 2006). In particular, understanding the mode-specificity in reaction dynamics of open-shell atoms, free radicals, molecules, atomic and molecular ions, under hyper-thermal, thermal, and sub-thermal conditions is of fundamental importance for catalysis, plasmas, photodynamics as well as interstellar, and low-temperature chemistry (see for instance Aquilanti et al., 2005; Chang et al., 2013; Li et al., 2014; Rösch et al., 2014; Balucani et al., 2015).

The possibility of aligning or orienting molecules by collisions in gaseous streams may also have some implications in unraveling the origin of chiral discrimination and chiral selectivity emerging in vortices formed both in the liquid and in the gas phase (Lombardi and Palazzetti, 2018, and references therein; Su et al., 2013).

On the basis of the experimental findings, achieved in the last 25 years by the authors, it is proper to distinguish:

*Molecular alignment determined by weak van der Waals forces:* It arises as a combined effect of several elastic/inelastic collisions occurring along preferential directions in environments where anisotropic velocity distributions are operative;

*Molecular orientation controlled by anisotropic intermolecular forces of intermediate strength*: Such phenomenon manifests even during single collision events, when the molecules are in low rotational states;

*Molecular orientation induced by anisotropic intermolecular forces of high strength*: It becomes dominant in each collision event under an ample variety of conditions.

The abovementioned classification is proposed on the basis of the results obtained exploiting different but complementary experimental techniques, in the Perugia and Trento laboratories, as well as an integrated experimental-theoretical approach. This paper focuses on selected results highlighting the role of molecular polarization, induced in a natural way by weak, intermediate and strongly anisotropic forces, on the reaction stereo-dynamics under a variety of conditions, including those of applied interest.

## Results and Discussion

### Molecular Alignment by Weak Anisotropic Forces

**Figure 1:**
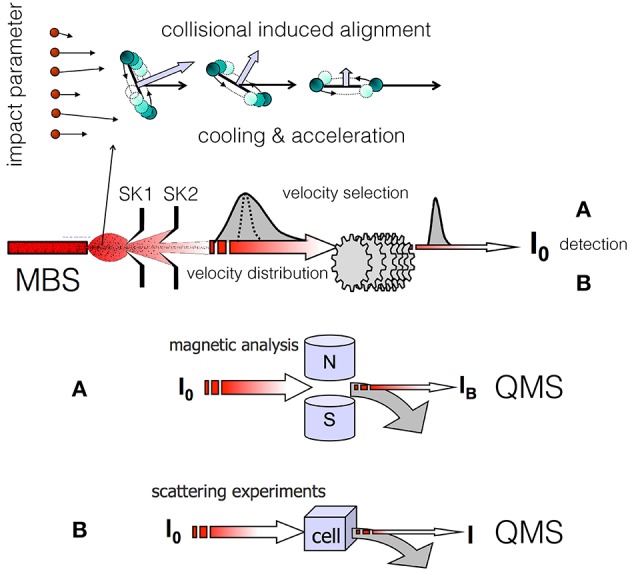
Illustration of dynamical processes (focusing in the forward direction of the expansion, bending of the rotational plane, rotational relaxation, acceleration) that a diatomic molecule, seeded in a lighter carrier gas, experiences during the formation of a supersonic molecular beam (MBS, Molecular Beam Source; SK1 and SK2, skimmers; QMS, quadrupole mass spectrometer). In this conceptual scheme a common MBS and velocity selection is exploited for the successive (A) magnetic analysis or (B) scattering experiment. In the magnetic analysis (A) the MB transmittance I_B_/I_0_ varies with the molecular velocity and paramagnetism. In the scattering experiments (B) the beam attenuation I/I_0_ varies with the molecular velocity and intermolecular forces that drive the scattering.

Molecular alignment induced by weak anisotropic van der Waals (vdW) forces emerges in supersonic expansions leading to the formation of *seeded molecular beams*, where hundreds of collisions between seeded molecules and lighter (hence faster) carrier atoms occur preferentially in the forward direction of the expansion. Value and direction of the relative collision velocity, also defined as *velocity slip*, play a crucial role in determining important selectivities in the involved elastic and inelastic collisions. To correctly identify the microscopic phenomena, it is useful to distinguish two different regions of the expansion zone. The first one is confined in the proximity of the source nozzle, where the *velocity slip* and *gas density* are both sufficiently high to promote, in addition to many-body elastic events, also inelastic collisions leading to both molecular rotational excitation and relaxation. The second zone is localized at larger distances from the nozzle, where *gas density* and *velocity slip* exhibit decreased values, and only elastic and inelastic processes at low energy (rotational relaxation) can occur. In the last region, where only two-body collisions are present, the promoted microscopic events can be classified in terms of the orbital angular momenta (or impact parameters *b* in a classical picture) involved (see the upper panel of Figure 1). Hence, the degree of achieved molecular alignment is expected to depend on the geometric features of the nozzle, on the pressures used in the source and on the resolution conditions adopted in the experiments. We have found that polarization effects are more evident when the angular cone probed around the beam axis becomes narrower, suggesting that the observed phenomena arise from marked stereodynamical effects (Pirani et al., 2001, 2003).

In the Perugia apparatus, two different experiments have been exploited as probes of the alignment in the produced molecular beams [see (A,B) in Figure 1], as detailed in (Aquilanti et al., 1994, 1995a,b, 1997). The probe experiments were performed at a considerable distance from the beam source and after a detailed velocity selection of the formed molecular beam (MB). The first experiment, applicable exclusively to paramagnetic molecules, exploits beam transmittance measurements across a Stern-Gerlach magnetic selector. This device probes the magnetic sublevel population of the molecules in the achieved final states and velocity. The second experiment, of more general applicability, involves measurements of beam intensity attenuation by collisions with an atomic or molecular target. The attenuation value, depending on the strength of the intermolecular interaction driving the scattering, is expected to vary with the relative orientation of the two colliding partners, hence on the helicity states populated by the projectile molecules.

**Figure 2:**
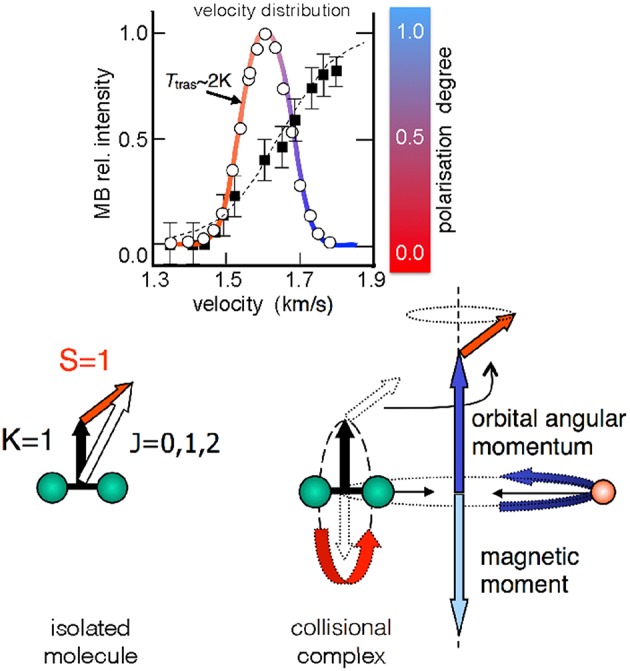
**Upper** relative O_2_ intensity (open circles) as a function of the molecular velocity for a He seeded supersonic beam (with 2.5% O_2_ in He). The fitted velocity distribution (colored continuous line) gives a translational temperature of ~2 K. Black squares represent the molecular polarization degree as quantitatively obtained in Aquilanti et al. (1994). The color code (red = no alignment, blue = high alignment) is a qualitative aid for the eye, also employed in the next figure (Figure 3). **Lower** angular momentum coupling for the ^16^O^16^O diatomic molecule in the ground ^3^∑_g_ electronic state. The spin-rotational sublevels, relevant for cold supersonic beams (T_tras_ of few Kelvin), are indicated. In the right side of the panel the case of a collision complex of O_2_ with a rare gas is represented. Herein, the electron spin is decoupled from the rotational angular momentum (see text).

The O_2_ molecule is an open shell species with total spin quantum number S = 1. The magnetic analysis performed on O_2_ seeded beams showed that molecules achieve a high and anomalous paramagnetism, related to a non-statistical distribution of their magnetic sublevels. The paramagnetic degree is found to increase with the final speed reached by the molecules within the same velocity distribution, and with the pressure employed in the source (Aquilanti et al., 1994, 1995a). In other words, molecules in a supersonic MB are preferentially confined in the *edge-on* configuration (upper panel, Figure 1, where the formation of zero helicity state is depicted), and the faster ones exhibit the highest polarization degree (upper panel, Figure 2). Crucial for the interpretation of experimental findings is to recall that the rotation-electronic angular momentum coupling for O_2_ is best described by Hund's case *b*. Besides, O_2_ molecules in seeded beams nearly exclusively populate the ground rotational state defined by the quantum number K = 1 and the associated angular momentum **K** couples with **S** to give the well-known spin-rotational sublevels, identified by J (**J=K+S**), as indicated in Figure 2. The analysis of the O_2_ beam paramagnetism, based on a non-statistical distribution of spin-rotation sublevels, suggested that the fastest molecules achieve a high simultaneous/combined polarization of **K** and **S** (Aquilanti et al., 1999).

The same analysis provided an average rotational alignment of O_2_ and a source pressure dependence similar to those from previous experiments on closed-shell molecules (see Aquilanti et al., 1995a and references therein). However, an open question concerns the microscopic mechanism leading to the simultaneous alignment of **K** and **S**. In particular, the focus is on the behavior of the electronic spin **S** that couples with other angular momenta exclusively via magnetic interactions. During a scattering event leading to a collision complex (Figure 2), the intermolecular electric field strength is sufficient to decouple **K** from **S**, and the field anisotropy (or gradient) tends to form states with zero helicity. Under such conditions, the single quantization axis for **S** is the direction of the orbital angular momentum of the collision complex. After the collision, **K** couples again with **S** and a polarization transfer between **K** and **S** can occur. Although observed in other phenomena (Ramsey, 1955; Bhaskar et al., 1982; Happer et al., 1984; Sofikitis et al., 2007) involving rotational, nuclear and electronic spin angular momentum couplings, probably all implications of the polarization transfer on the molecular dynamics are still not fully understood.

**Figure 3:**
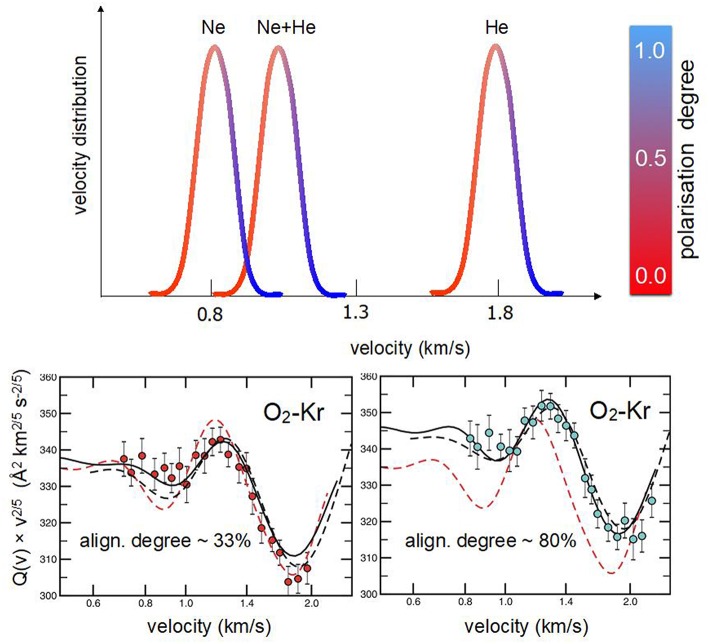
**Upper** O_2_ velocity distributions in supersonic beams seeded (2.5%) in Ne, Ne:He mixture, and He. The color code is an indication of the molecular alignment degree in the beam (see Figure 2). **Lower** integral cross sections *Q(v)* for elastic scattering of O_2_ with Kr, with their dependence on the MB velocity *v*, are shown. Data, reported as *Q(v)v*^2/5^ in ordinate scale, are plotted as a function of the selected velocity, in logarithmic scale on the abscissa axis, to emphasize the glory pattern due to the quantum interference (Aquilanti et al., 1998). In the **Left hand** results (red circles) correspond to molecules selected in the slow tail of the velocity distribution (i.e., no alignment related to a speed ratio of 0.95). The **Right hand** depicts the case of O_2_ molecules selected in the fast front (head) of the velocity distribution (high molecular polarization associated to a speed ratio of 1.10).

The investigation of O_2_ in several seeded beams showed that the achieved alignment degree is nearly independent of the type of lighter carrier species and that a proper scaling factor is the *reduced speed* (or *speed ratio*), defined as the ratio between the selected molecular velocity and the peak velocity of the MB. Therefore, merely exploiting the velocity selection technique, it has been possible to sample, in a controlled way, molecules flying at the same speed but having a different alignment degree (Aquilanti et al., 1994, 1995a). Accordingly, it was possible to measure, at the same collision velocity, cross-section anisotropy arising from the different alignment degree of projectile molecules with respect to the collision direction (see Figure 3). In particular, using Kr and Xe atoms as targets, scattering cross sections have been measured (Aquilanti et al., 1998) selecting specific *speed ratios*, related to molecules flying in the tail, peak, near the head and in the head of several seeded beams. This sampling allowed to quantify:

- the velocity dependence of the cross section for projectile molecules flying with the same *speed ratio* and
- the cross section anisotropy, at fixed collision velocity, due to a change in the *speed ratio* (see Figure 3), hence of the molecular alignment degree.

The combined analysis of beam paramagnetism and scattering experiments confirmed the dependence of the O_2_ molecular alignment on the *speed ratio* and allowed to obtain intermolecular potential energy surfaces (PESs) in the anisotropic and weakly interacting O_2_-Kr and O_2_-Xe systems.

In a similar manner, by exploiting the combination of velocity selection with scattering experiments, the cross section anisotropy in collisions of diamagnetic N_2_ projectiles with Xe target were measured (Aquilanti et al., 1997). Correspondence of the measured anisotropy has also been found (Vattuone et al., 2010) with literature data on the scattering of oriented NO molecules by Xe (Reuss, 1975; Thuis et al., 1979). Such authors controlled the NO orientation by the external electric fields of an hexapole. Moreover, in the N_2_ experiments, the use of a defined PES allowed extracting information on the molecular alignment degree, that exhibits a speed ratio dependence very similar to that of O_2_.

Within a Trento-Perugia collaboration, a combined study making use of scattering experiments and spectroscopic probes has been performed on seeded beams of hydrocarbon molecules (Pirani et al., 2001, 2003). The use of two different and independent techniques, probing two different observables related to molecular orientation, has provided interesting insights in the alignment processes of such molecules. The scattering technique, already described, uses the total integral cross section dependence on the relative orientation of the projectile molecule with respect to the target one. The spectroscopic technique is based on the dependence of the intensity of some rovibrational transitions on the relative orientation between the transition dipole moment of the molecule and the light polarization vector.

Benzene has been the first examined system (Pirani et al., 2001, 2003). It is a planar molecule exhibiting a strong dependence of the scattering cross section on the orientation of the molecular plane with respect to the velocity direction. The spectroscopic experiment probed a C-H stretching transition whose dipole moment lies in the molecular plane. Hence, by analyzing the absorption intensity as a function of the angle between the light polarization vector and the velocity vector of the molecule, the amount of anisotropy in the molecular orientation states was quantified.

In this investigation both techniques gave the same indication: after supersonic expansion there is a net deviation of the scattering and optical observables from the values expected in case of isotropic distribution of orientation (i.e., *flying*) modes: the states with molecular plane parallel to the velocity direction (*frisbees*) are considerably more probable than those having the molecular plane perpendicular to it (*flywheels*).

The combined experiment gave also relevant clues on final velocity dependence, previously discussed, and on the angular cone amplitude around the beam axis sampled after the supersonic expansion, confirming that, also for large molecules, the alignment process is dependent on stereo-dynamical processes due to collisions during beam expansion.

### Molecular Orientation by Intermediate Strength Forces

In the last 10 years, particular attention has been addressed to the scattering of water molecules by several targets (Cappelletti et al., 2012) and to develop model potentials describing the interaction of H_2_O molecules in neutral and ionic clusters (Albertí et al., 2009). Water is a polar species having a permanent electric dipole moment equal to 1.85 Debye, and it exhibits electronic polarizability very close to that of Ar. MB scattering experiments with a series of hydrogenated polar molecules have been performed in order to systematically investigate the phenomenology associated to anisotropy effects in collisions of polar hydrogenated molecules, as a function of the product of their permanent electric dipoles. Integral cross-section *Q(v)* values, measured for the D_2_O-D_2_O, D_2_O-ND_3_, D_2_O-H_2_S, and ND_3_-H_2_S colliding pairs, have been reported and discussed (Roncaratti et al., 2014a,b) in comparison with those of reference systems Ar-Ar, Ar-Kr, Ar-Xe, and Kr-Xe.

The choice of reference systems has been suggested by the following similarities in the polarizability values: Ar (1.6 Å^3^) and water (1.5 Å^3^), Kr (2.5 Å^3^) and ammonia (2.2 Å^3^), Xe (4.0 Å^3^), and hydrogen sulfide (3.8 Å^3^). The isotropic polarizability, related to the particle size and to the probability of induced electric dipole formation, represents a proper scaling factor of both average size repulsion and of dispersion/induction attraction. It should be noted that, when dealing with molecules, the overall polarizability includes contributions from the constituent atoms as well as from bonds. Therefore, each pair of investigated molecular and atomic reference systems is expected to exhibit the same isotropic van der Waals interaction. Measured scattering results clearly indicate that all systems formed by two hydrogenated polar molecules exhibit much larger cross-sections *Q(v)*, well outside the experimental uncertainty, than those of the corresponding reference atomic ones. In particular, we evaluated that the cross section ratios of the polar pairs vs. the corresponding reference systems are (on average) ~2.5 for D_2_O-D_2_O, ~1.7 for D_2_O-ND_3_, ~1.4 for D_2_O-H_2_S, and ~1.2 for ND_3_-H_2_S. These *Q(v)* ratios show a clear linear dependence on the product of the dipole moments of the colliding partners, suggesting an appreciable role of the electrostatic component of the intermolecular potential on the investigated experimental observables (Roncaratti et al., 2014a,b). A negligible role is expected for collisions with randomly oriented partners, since the electrostatic component vanishes. This is a clear evidence that, during collisions between polar molecules, the latter do not maintain random orientations and are not freely rotating, but tend to be trapped in *pendular states*, where they experience a much stronger interaction than that obtained by averaging over all relative configurations.

**Figure 4:**
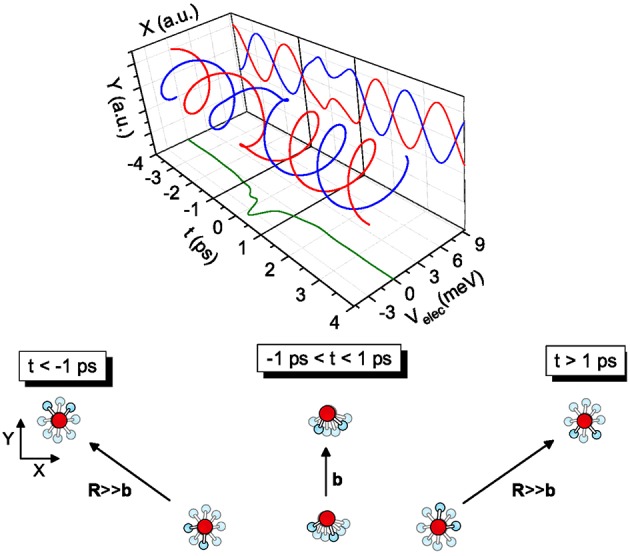
Examples of coplanar molecular collisions—The case of two water (D_2_O) molecules colliding at relative velocity g = 1.0 km/s and impact parameter *b* = 10 Å is illustrated. The system before the collision is defined by a negative *t*, after the collision by a positive *t* and at the turning point, i.e., the distance of closest approach, by *t* = *0*. The elastic collision between molecules both in *J* = 1 is depicted. The coupling induces a local modification of the molecular modes, clearly evidenced when the relative motion of the two dipoles is projected on the Y-t plane. The collision complex dynamics is driven by an effective electrostatic dipole-dipole interaction. Most part of its influence manifests in the time scale from −1 to +1 ps.

The transition from *free rotors* to *pendular states* is promoted by the coupling between the molecular permanent dipole moments within the field gradient due to the anisotropic intermolecular potential. The phenomenon, occurring in the timescale of ps (i.e., similar to the time required for a collision at thermal energies) couples more effectively molecules with very similar rotational periods and populating low lying rotational states (Roncaratti et al., 2014a,b). Under such most favorable conditions, the coupling originates the so-called *synchronized dance of water molecules* (see Figure 4), a phenomenon crucial to describe the passage of water molecules in carbon nanotubes and cellular channels.

Further experimental investigations, integrated by advanced theoretical calculations, have been also extended to mixed (prototype) systems, formed by polar molecules—noble gas atom pair, to characterize selectivities in the formation of weak intermolecular hydrogen bond (Cappelletti et al., 2012).

### Molecular Orientation by Strong Intermolecular Forces

In the case of ion-molecule reactions, alignment/orientation is a general phenomenon due to the electric field generated by the charged particle. Stereo-dynamic effects related to long-range anisotropic interactions have been observed in several systems, with different outcomes on reaction probability. A pertinent example is given by the (Ar-N_2_)^+^ system, whereby a reasonable description of the charge-transfer dynamics can only be achieved by accounting for the spin–orbit interaction, the molecular anisotropy and the electronic anisotropy related to the open-shell nature of Ar^+^ (Candori et al., 2001, 2003).

In general, when alignment/orientation drives the collision complex into the most appropriate configurations for reaction, an enhancement of reactivity is possible. It is the case of the ${\text{H}}_{2}^{+}$ + H_2_ → ${\text{H}}_{3}^{+}$ + H reaction, for which an enhancement of the rate coefficient with respect to the classical Langevin-capture behavior at low collision energies has been attributed to anisotropic modification of the long-range scattering potential due to interaction between the ${\text{H}}_{2}^{+}$ charge and the rotational quadrupole moment of the ground state of ortho-H_2_ (Allmendinger et al., 2016).

On the other hand, when long-range interaction potentials reorient the reacting couple, either in a non-reactive or in a configuration unfavorable for reaction, the overall reaction probability will be suppressed, as in the case of the H-atom transfer reaction between H_2_ and H_2_O^+^. In the latter system, the most attractive orientation, governed by charge and dipole-quadrupole/induced multipole interactions, is not the most favorable for H atom transfer. Thus reorientation of H_2_O^+^, facilitated by rotational excitation, is necessary to promote reactivity (Li et al., 2014). Hence, rate constants can show an Arrhenius dependence (i.e., a positive dependence on T) even in the case of barrierless and exothermic processes, as observed in the reactions of Ar^+^ and ${\text{N}}_{2}^{+}$ ions with diatomic interhalogens ICl and ClF (Shuman et al., 2017).

Differences in the long-range ion-molecule interaction potentials are also at the basis of the different bimolecular reactivity observed by different rotational isomers (conformers) of a polyatomic molecule in the gas phase. By elegantly exploiting an experimental technique based on the spatial separation of conformers having significantly different electric dipole moments in a MB via electrostatic deflection (i.e., the use of inhomogeneous electric fields), the specific chemical reactivity of two conformers of 3-aminophenol with cold Ca^+^ ions in a Coulomb crystal was observed (Chang et al., 2013; Rösch et al., 2014), thus demonstrating the possibility of controlling reactivity through selection of conformational states.

A recent investigation from our laboratories focused on the study of some ion-molecule reactions of relevance to assess the competition and balance of phenomena occurring in many gaseous and plasma environments, ranging from the ionospheres of planets and the interstellar medium (Balucani et al., 2015) (low temperatures) to laboratory plasmas for technological applications (much higher temperatures). In particular, collisions with He^+^ are an important pathway for the decomposition of “complex organic molecules” (COMs, i.e., molecules containing at least six atoms) in various astronomical environments (Balucani et al., 2015; Ascenzi et al., 2019). Since dimethyl ether (DME) and methyl formate (MF) are among the most abundant COMs, experiments have been performed on the reactivity of He^+^ ions with such neutrals, using a Guided Ion Beam Mass Spectrometer, which allows measurements of reactive cross sections and branching ratio (BR) as a function of the collision energy (Cernuto et al., 2017, 2018). Due to the large dipole moments exhibited by the neutral collision partners (1.30 Debye for DME and 1.77 Debye for MF) the studied systems present large interaction anisotropies that can induce strong stereodynamical effects and influence the outcome of reactive collisions. The experimental evidence is that the electron exchange processes are completely dissociative, leading to extensive fragmentation of the neutral partner, and cross section trends with collision energies are at odds with those expected from simple *capture models*. By investigating the nature of the non-adiabatic transitions between the reactant and product potential energy surfaces using an improved Landau-Zener model, we were able to identify three critical elements at the basis of such discrepancy:

- the strong anisotropy of the entrance PES (see section PES anisotropy)
- the crossing positions among entrance and exit PESs (see section Crossings among entrance and exit diabatic PESs)
- the symmetry of the electron density distribution of the inner valence molecular orbitals of DME and MF involved in the electron transfer to He^+^ (see section Symmetry of the electron density distributions).

#### PES Anisotropy

**Figure 5:**
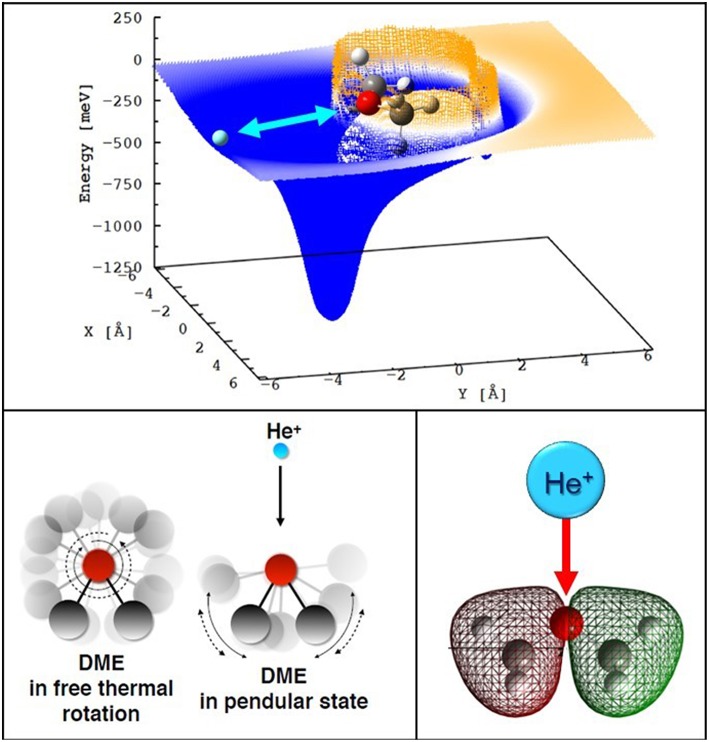
**Top** section of the PES for the entrance channel of the He^+^-DME system (the cation is confined in the C-O-C plane). **Bottom left** a sketch of the formation of a *pendular state* while He^+^ ions approach a freely rotating DME molecule. **Bottom right:** pictorial view of minimal overlap between the spherically symmetric atomic orbital of He^+^ and the inner valence orbital of DME from which the electron is extracted.

For both DME and MF the interaction anisotropy in the entrance channels is such that one (two for the MF case) deep potential wells (with depth in the range 1.3–1.7 eV) are present for selected configurations: namely when the He^+^ ion approaches the molecule from the O atom side, on the plane defined by the C atoms and the ethereous O atom (see Figure 5, top panel). As a consequence, the reaction dynamics is substantially limited to a few geometries confined around the most stable configurations of the collision complex. The rather large interaction anisotropy induces pronounced orientations of the polar neutrals in the electric field generated by He^+^, which are mostly operative at low collision energies. While the neutrals are free to rotate at large distances, as the colliding partners come closer, the rotation of the polar molecule becomes partially hindered by the intermolecular electric field gradient associated with the interaction anisotropy. Hence, at short distances, the collision complex is confined within *pendular states*, a particular case of bending motion (see Figure 5, bottom left panel). Such orientation effects can influence the dynamic of the charge-exchange process by channeling most of the neutral molecules in narrow angular cones confined around the most attractive configurations of the interacting systems. Similar effects have been also observed at the interface between the gas and liquid phase for solutions containing cations and anions (Gisler and Nesbitt, 2012).

#### Crossings Among Entrance and Exit Diabatic PESs

Due to the large differences in ionization energy (IE) between He (IE = 24.59 eV) and DME (IE = 10.025 eV) or MF (10.835 eV), analysis of the interaction PESs shows that the (diabatic) reactant surfaces do not cross the product surfaces correlating asymptotically with the ground state of DME^+^ and MF^+^. Thus, He^+^ captures an electron from an inner valence orbital of the neutral molecules, forming the molecular cation in a highly excited state, that quickly dissociates.

#### Symmetry of the Electron Density Distributions

The symmetry of the electron density distribution of the molecular orbitals from which the electron is removed turns out to be a further major point affecting the probability of electron transfer to He^+^, since it affects the overlap integral between the orbitals involved in the electron exchange. In both DME and MF cases, at least one of the molecular orbitals that are expected, in terms of crossing positions, to give the most significant contribution to charge transfer, presents a small overlap with the spherically symmetric atomic orbital of He^+^(^2^S_1/2_), as pictorially shown in the bottom right panel of Figure 5. This effect originates the paradox that the most attractive geometry is the least efficient for charge transfer, and the reactions are increasingly driven by the Coriolis coupling (i.e., the coupling between the rotational angular momentum of nuclei in the collision complex and the orbital angular momentum of the electron) rather than by orbital overlap. Using such a combined experimental and theoretical methodology we have been able to provide new values for the temperature dependent rate coefficients and branching ratios of the reactions of He^+^ with the two important COM, dimethyl ether and methyl formate (MF). Our results will be relevant for a correct modeling of the chemical kinetics in various regions of the interstellar space, such as prestellar cores and hot corinos (Ascenzi et al., 2019).

In conclusion, all the experimental findings, characterized in several experiments carried out within the Perugia-Trento collaboration, can be rationalized in a unifying picture that considers the sterodynamics of gas phase collisions controlled by anisotropic forces of different strength.

## Author Contributions

FP and DA wrote the first draft of the manuscript. PT, MS, and DC wrote sections of the manuscript. All authors contributed to manuscript revision, read and approved the submitted version and contributed conception and design of the study.

### Conflict of Interest Statement

The authors declare that the research was conducted in the absence of any commercial or financial relationships that could be construed as a potential conflict of interest.
